# Effects of 6-Benzyladenine (6-BA) on the Filling Process of Maize Grains Placed at Different Ear Positions under High Planting Density

**DOI:** 10.3390/plants12203590

**Published:** 2023-10-16

**Authors:** Tao Yu, Yuning Xin, Peng Liu

**Affiliations:** 1 College of Plant Protection, Shandong Agricultural University, Taian 271018, China; yutaosdnd@sdau.edu.cn; 2 College of Agronomy, Shandong Agricultural University, Taian 271018, China; xinyuningsdau@126.com

**Keywords:** maize, 6-benzyladenine, grain weight, grain position, grain filling, starch synthesis, hormone

## Abstract

Increasing grain weight under dense planting conditions can further improve maize yield. 6-BA is known to be involved in regulating grain development and influencing grain weight. Maize grain development is closely linked to starch accumulation and hormone levels. In this work, the effects of applying 6-BA at the flowering stage under high density on the grain filling characteristics, starch content, starch synthesis critical enzyme activity, and endogenous hormones levels of maize grains (including inferior grains (IGs) and superior grains (SGs)) of two high-yielding summer maize varieties widely cultivated in China were investigated. The findings indicated that applying 6-BA significantly improved maize yield compared to the control, mainly as a result of increased grain weight due to a faster grain filling rate. Additionally, the activities of enzymes associated with starch synthesis, including sucrose synthase (SuSy), ADP-glucose pyrophosphorylase (AGPase), granule-bound starch synthase (GBSS), soluble starch synthase (SSS), and starch branching enzyme (SBE), were all increased following 6-BA application, thus facilitating starch accumulation in the grains. Applying 6-BA also increased the zeatin riboside (ZR), indole-3-acetic acid (IAA), and abscisic acid (ABA) levels, and reduced the gibberellin (GA_3_) level in the grains, which further improved grain filling. It is worth noting that IG had a poorer filling process than SG, possibly due to the low activities of critical enzymes for starch synthesis and imbalanced endogenous hormones levels. However, IG responded more strongly to exogenous 6-BA than SG. It appears that applying 6-BA is beneficial in improving filling characteristics, promoting starch accumulation by enhancing the activities of critical enzymes for starch synthesis, and altering endogenous hormones levels in the grains, thus improving grain filling and increasing the final grain weight and yield of maize grown under crowded conditions. These results provide theoretical and technical support for the further utilization of exogenous hormones in high-density maize production.

## 1. Introduction

Maize is a globally important food crop, so ensuring a high and consistent maize yield is a strategic priority for global food security [1,2]. Among the yield components of maize, grain weight plays a primary role in determining overall yield, following ear number and grains per ear [3,4]. Higher planting densities are now widely used worldwide to increase maize yield by maximizing energy and nutrient use [5,6,7]. However, grain weight has been reduced due to the intense competition between plants for survival resources under high-density conditions, limiting the potential for high maize yield [8,9,10]. Therefore, increasing grain weight under dense planting conditions remains a challenging issue in modern production systems to further improve maize yield.

Varietal characteristics, climatic conditions (e.g., temperature, rainfall, and light), and cultivation practices (e.g., planting density, fertilizer application, and irrigation) all have a significant effect on grain weight [11,12,13]. Simultaneously, maize grains are usually divided into inferior grains (IGs) and superior grains (SGs) according to their position on the ear, with the former typically placed in the upper part of the ear and the latter typically in the middle and lower part of the ear [14]. Maize IG was smaller in size and had lower weight compared to SG during the grain filling process [15,16]. These differences are often seen in other cereal crops such as rice [17] and wheat [18]. Inadequate grain filling of IG is considered to be a major problem limiting the high yield potential of the crop [19]. Additionally, grain weight was jointly influenced by the interaction between grain filling rate and duration, which are dynamic variables within the grain growth [20]. This means that substantial improvements in grain weight can be achieved by increasing the grain filling rate while maintaining duration.

Grain weight is strongly influenced by the biosynthesis and accumulation of starch, which serves as the main storage substance in maize grains. Starch formation is closely linked to the activities of critical enzymes such as sucrose synthase (SuSy), ADP-glucose pyrophosphorylase (AGPase), granule-bound starch synthase (GBSS), soluble starch synthase (SSS), and starch branching enzyme (SBE) [21,22]. Previous studies have shown that appropriate cultural practices such as slow-release fertilizer application, increased nitrogen supply, and chemical control can stimulate starch formation by increasing the activities of these enzymes, thereby improving maize grain filling [23,24]. It can be seen that variations in the activities of these critical enzymes for starch synthesis can regulate grain filling by affecting starch formation.

Grain development in maize, wheat, and rice is dependent on plant hormones such as zeatin riboside (ZR), indole-3-acetic acid (IAA), abscisic acid (ABA), and gibberellin (GA_3_) [25,26,27]. For example, high levels of ZR and IAA during the early stages of grain development can promote endosperm cell proliferation, thereby increasing sink capacity [28,29]. ABA and GA_3_ also control grain development by regulating the activity of numerous metabolic enzymes, hormone levels, and storage material accumulation [30,31,32]. In wheat grains, ZR, ABA, and IAA levels displayed a positive and significant relationship with grain filling rate [33]. In addition, studies in rice [34] and wheat [35] showed that IAA, ZR, and ABA levels were significantly higher in SG than in IG during grain development, suggesting that an imbalance in endogenous hormones levels is an important cause of the developmental differences between IG and SG. It is clear from these studies that variations in endogenous hormones levels in grains significantly affect grain filling and weight.

Several studies have conclusively shown that the use of exogenous hormones in field management can regulate endogenous hormones signals and various physiological and biochemical processes to promote grain development [36,37,38]. 6-BA, a synthetically produced cytokinin-like growth regulator, has a key regulatory function in plant growth and plant defense against environmental stress [39]. For example, the application of 6-BA at appropriate concentrations can increase leaf chlorophyll levels, promote photosynthesis, delay leaf senescence, and thus increase grain yield [40]. Gao et al. showed that applying 6-BA at the tasseling stage improved grain filling by enhancing source and sink capabilities, resulting in higher maize yield [41]. In addition, 6-BA can improve crop resistance to stresses such as cold, drought, salt, and waterlogging [42,43,44,45]. However, little attention has been paid to the effects of applying 6-BA on filling characteristics, starch content, starch synthesis critical enzyme activities, endogenous hormones levels, etc., of maize grains under high-density conditions. In particular, the effect on the filling process of maize grains at different ear positions is still unclear. Therefore, this work aimed to examine the effects of applying 6-BA on the filling process of maize grains (including IG and SG located at different ear positions) under high planting density. This study will provide both theoretical and practical guidance for the chemical regulation of maize under dense planting conditions.

## 2. Results

### 2.1. Yield and Yield Components 

Compared to the control, the 1000-grain weight and yield of both varieties increased significantly after the 6-BA application (Table 1). The two-year average 1000-grain weight and yield of DH605 increased markedly by 7.82% and 8.11%, respectively, while those of ZD958 increased markedly by 6.02% and 6.69%, respectively, following 6-BA treatment compared to the control. Maize yield components include grain weight, ear number, and grains per ear. However, no significant differences in the ear number or grains per ear were observed between the control and 6-BA treatments for either variety. This suggested that the improvement in grain weight following the 6-BA application was the main reason for the increase in maize yield.

### 2.2. Grain Filling Process

**Figure 1:**
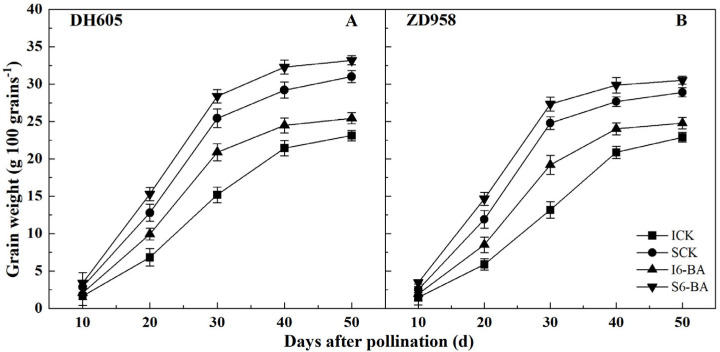
Effects of applying 6-BA on grain filling process (2022). (**A**), The grain weight in DH605; (**B**), The grain weight in ZD958. ICK and I6-BA correspond to inferior grains treated with water and 6-BA, respectively. SCK and S6-BA correspond to superior grains treated with water and 6-BA, respectively. Means and standard errors are presented from three replications. Multiple comparisons of treatment means within a growth stage were performed using Duncan’s test (*p* = 0.05).

The patterns of grain weight variation were consistent across all treatments (Figure 1). At each sampling period, IG had a lower grain weight than SG in both varieties. Applying 6-BA increased grain weight compared to the control, with IG responding more to exogenous 6-BA than SG in both varieties. At 50 DAP, the grain weight of IG increased by 9.97% and 8.32%, while that of SG increased by 7.03% and 5.56% in DH605 and ZD958, respectively, after 6-BA application compared to the control.

Grain weight of achieving maximum grain filling rate (W_max_), maximum grain filling rate (G_max_), average grain filling rate (G_ave_), and active grain filling duration (P) are important parameters reflecting the grain filling process. Compared to SG, IG of both varieties had poor grain filling characteristics as indicated by a significant reduction in W_max_, G_max_, and G_ave_ (Table 2). However, P was not significantly different between IG and SG, indicating that the slow filling rate is the main reason for the poorer filling in IG. In both varieties, there was a significant increase in grain W_max_, G_max_, and G_ave_ following 6-BA treatment compared to the control, but no significant effect on P. W_max_, G_max_, and G_ave_ were more sensitive to exogenous 6-BA in IG than in SG. In DH605, 6-BA application significantly increased W_max_, G_max_, and G_ave_ by 16.62%, 19.18%, and 18.18% in IG and by 14.20%, 11.88%, and 13.33% in SG, respectively, compared to the control. In ZD958 after 6-BA application, W_max_, G_max_, and G_ave_ of IG were markedly increased by 8.20%, 14.63%, and 14.29%, respectively, while those of SG were markedly increased by 6.48%, 9.62%, and 9.68%, respectively, compared to the control. These results indicated that the 6-BA application mainly accelerated the grain filling rate to improve the filling process.

### 2.3. Grain Starch Content and Starch Synthesis Critical Enzyme Activity

#### 2.3.1. Starch Content 

**Figure 2:**
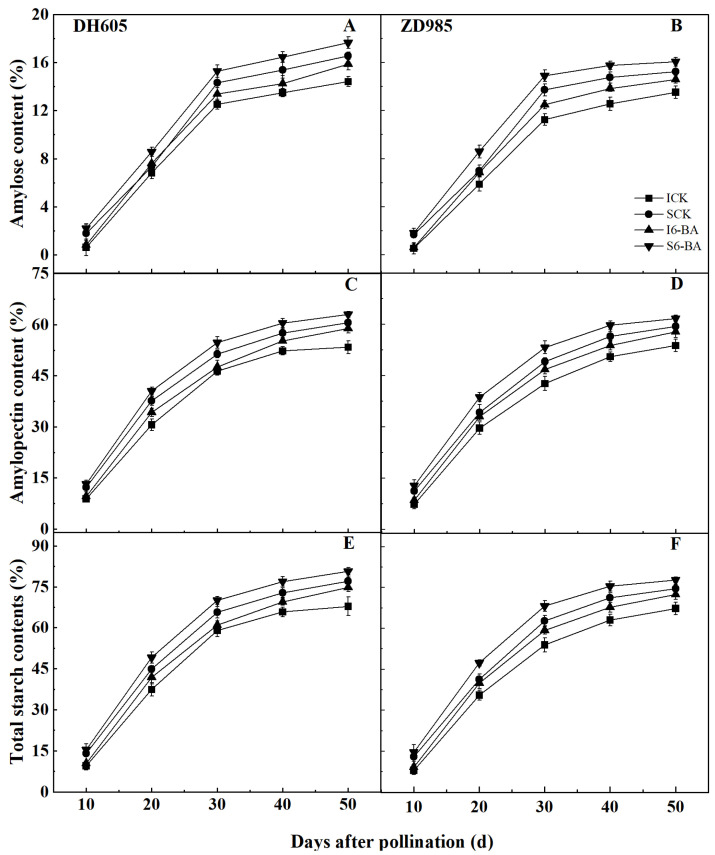
Effects of applying 6-BA on amylose, amylopectin, and total starch contents (2022). (**A**,**C**,**E**), The contents of amylose, amylopectin, and total starch in DH605 grains; (**B**,**D**,**F**), The contents of amylose, amylopectin, and total starch in ZD958 grains. ICK and I6-BA correspond to inferior grains treated with water and 6-BA, respectively. SCK and S6-BA correspond to superior grains treated with water and 6-BA, respectively. Means and standard errors are presented from three replications. Multiple comparisons of treatment means within a growth stage were performed using Duncan’s test (*p* = 0.05).

Starch, including amylose and amylopectin, is the main storage material in maize grains. Similar to grain weight, IG had significantly lower amylose, amylopectin, and total starch contents than SG in both varieties (Figure 2). These starch contents were all increased following 6-BA application compared to the control, and IG had a greater response to exogenous 6-BA than SG in both varieties. Compared to the control, in DH605 treated with 6-BA at 50 DAP, the amylose, amylopectin, and total starch contents of IG were markedly increased by 10.08%, 10.22%, and 10.19%, respectively, while those of SG were markedly increased by 6.67%, 4.16%, and 4.70%, respectively. In ZD958 at 50 DAP, applying 6-BA markedly improved the amylose, amylopectin, and total starch contents in IG by 7.97%, 7.47%, and 7.57% and in SG by 5.27%, 3.90%, and 4.18%, respectively, compared to the control.

#### 2.3.2. Starch Synthesis Critical Enzyme Activity 

**Figure 3:**
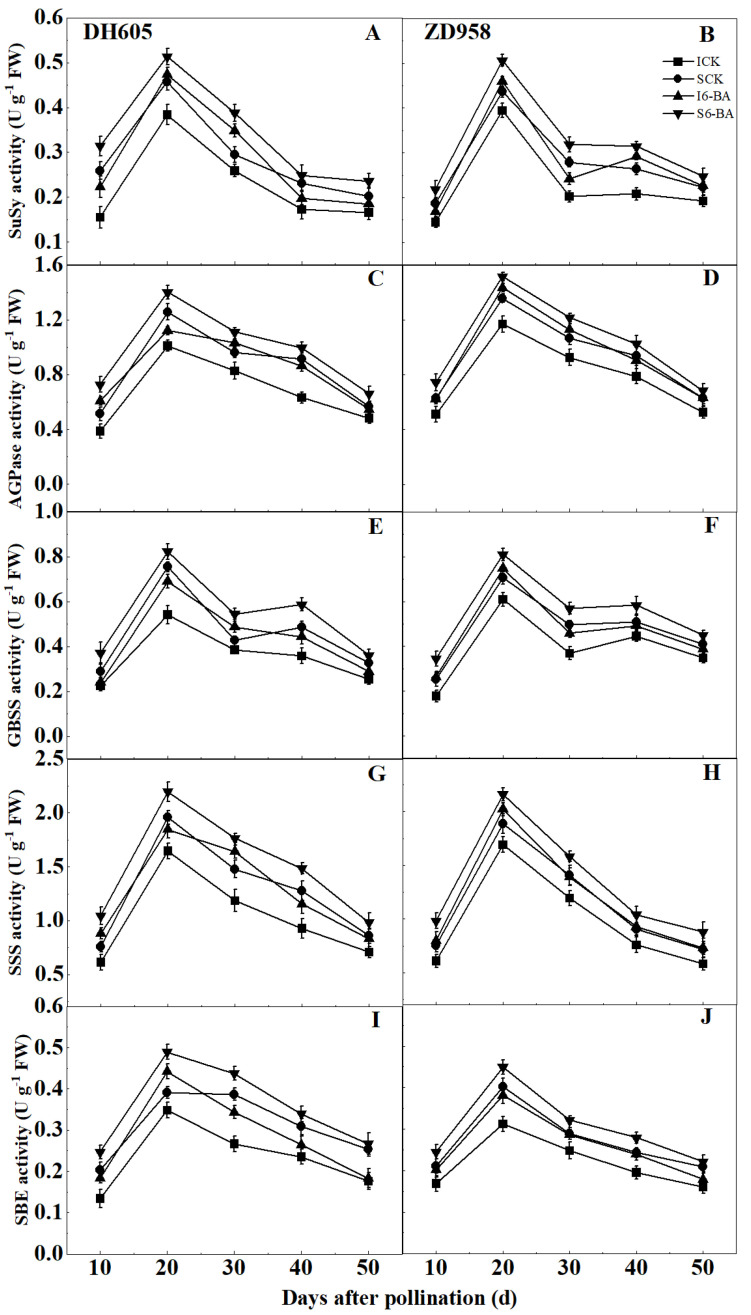
Effects of applying 6-BA on the activities of critical enzymes for starch synthesis (2022). (**A**,**C**,**E**,**G**,**I**), The activities of SuSy, AGPase, GBSS, SSS, and SBE in DH605 grains; (**B**,**D**,**F**,**H**,**J**), The activities of SuSy, AGPase, GBSS, SSS, and SBE in ZD958 grains. ICK and I6-BA correspond to inferior grains treated with water and 6-BA, respectively. SCK and S6-BA correspond to superior grains treated with water and 6-BA, respectively. SuSy, sucrose synthase; AGPase, ADP-glucose pyrophosphorylase; GBSS, granule-bound starch synthase; SSS, soluble starch synthase; SBE, starch branching enzyme. Means and standard errors are presented from three replications. Multiple comparisons of treatment means within a growth stage were performed using Duncan’s test (*p* = 0.05).

SuSy, AGPase, GBSS, SSS, and SBE activities were closely related to starch accumulation. They showed similar trends in all treatments (Figure 3). In both varieties, the above enzyme activities increased between 10 and 20 DAP, peaked at 20 DAP, and then declined. The activities of these enzymes were all lower in IG than in SG at each sampling period. The application of 6-BA showed a positive regulation of the activities of these enzymes compared to the control, and IG responded more strongly to exogenous 6-BA than SG in both varieties. In DH605, the average activities of SuSy within 10 to 30 DAP, AGPase within 10 to 40 DAP, GBSS within 20 to 40 DAP, SSS within 10 to 40 DAP, and SBE within 10 to 30 DAP were significantly enhanced by 33.83%, 32.35%, 25.89%, 29.58%, and 30.77% in IG and by 21.72%, 19.54%, 13.77%, 21.42%, and 19.71% in SG, respectively, after applying 6-BA compared to the control. In ZD958 after 6-BA application, the mean activities of SuSy from 20 to 40 DAP, AGPase from 10 to 30 DAP, GBSS from 10 to 30 DAP, SSS from 10 to 30 DAP, and SBE from 20 to 40 DAP of IG were markedly increased by 25.32%, 21.88%, 18.88%, 22.47%, and 17.90%, respectively, while those of SG were markedly increased by 11.47%, 14.71%, 16.68%, 18.73%, and 11.39%, respectively, compared to the control. These results suggested that the application of 6-BA could promote starch accumulation by increasing the activities of these enzymes.

### 2.4. Grain Endogenous Hormones

#### 2.4.1. ZR, IAA, and ABA Levels 

**Figure 4:**
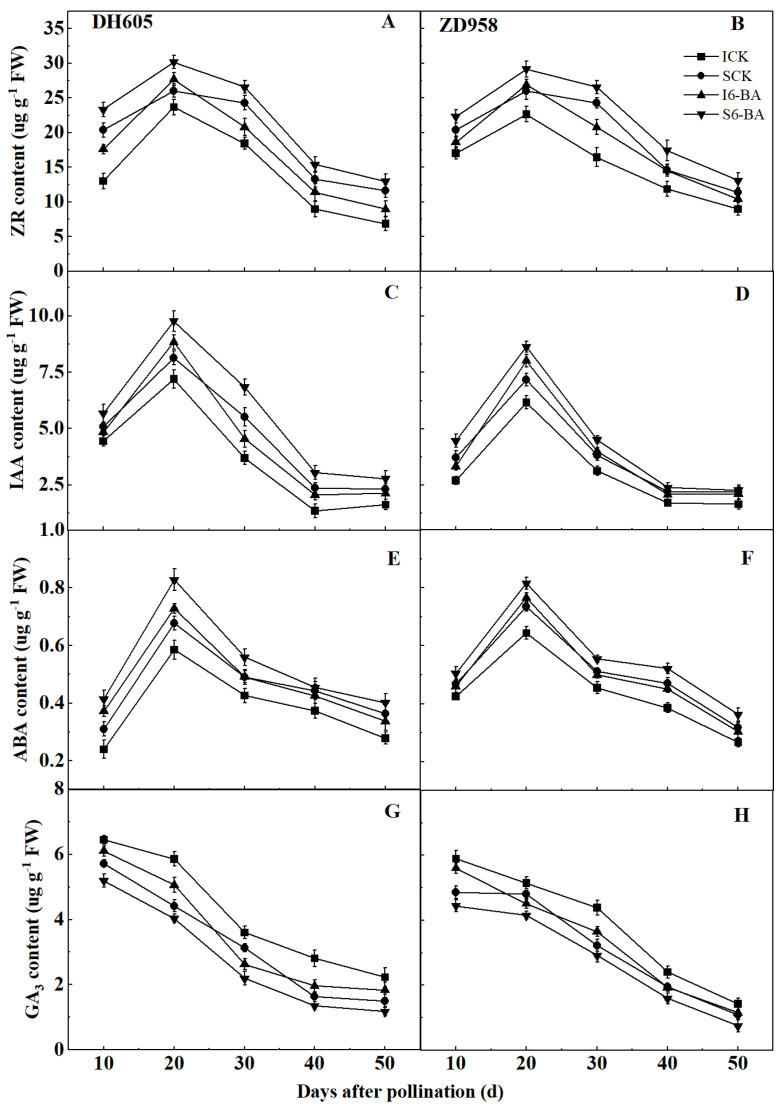
Effects of applying 6-BA on the levels of endogenous hormones (2022). (**A**,**C**,**E**,**G**), The levels of ZR, IAA, ABA, and GA_3_ in DH605 grains; (**B**,**D**,**F**,**H**), The levels of ZR, IAA, ABA, and GA_3_ in ZD958 grains. ICK and I6-BA correspond to inferior grains treated with water and 6-BA, respectively. SCK and S6-BA correspond to superior grains treated with water and 6-BA, respectively. ZR, zeatin riboside; IAA, indole-3-acetic acid; ABA, abscisic acid; GA_3_, gibberellin. Means and standard errors are presented from three replications. Multiple comparisons of treatment means within a growth stage were performed using Duncan’s test (*p* = 0.05).

Similar dynamic trends were observed for ZR, IAA, and ABA levels in all treatments (Figure 4A–F). These hormones levels first increased and then decreased between 10 and 50 DAP, reaching their highest values at 20 DAP, and were all lower in IG than in SG at each sampling period in both varieties. Applying 6-BA did not affect the trend of these hormones but increased the hormone levels in the grains compared to the control. These hormones showed a greater response to exogenous 6-BA in IG than in SG in both varieties. In DH605, the average levels of ZR from 10 to 30 DAP, IAA from 20 to 40 DAP, and ABA from 10 to 30 DAP were significantly increased by 21.66%, 32.11%, and 31.53% in IG and by 13.28%, 24.18%, and 23.20% in SG, respectively, after 6-BA application compared to the control. In ZD958 after applying 6-BA, the average levels of ZR from 20 to 40 DAP, IAA from 10 to 30 DAP, and ABA from 20 to 40 DAP of IG were significantly increased by 22.59%, 26.90%, and 15.27%, respectively, while those of SG were significantly increased by 13.58%, 19.44%, and 9.99%, respectively, compared to the control.

#### 2.4.2. GA_3_ Level 

Contrary to ZR, IAA, and ABA, the GA_3_ level followed a decreasing trend for all treatments and remained higher in IG compared to SG at each sampling period in both varieties (Figure 4G,H). When 6-BA was applied, a decrease in GA_3_ level was observed in IG and SG of DH605 and ZD958 between 10 and 40 DAP and 20 and 40 DAP, respectively, compared to the control. In addition, IG was found to be more responsive to exogenous 6-BA than SG. In DH605, the average GA_3_ level from 10 to 40 DAP of IG and SG was markedly reduced by 19.00% and 16.35%, respectively, after 6-BA application compared to the control. In ZD958, applying 6-BA significantly reduced the mean GA_3_ level from 20 to 40 DAP in IG and SG by 16.33% and 13.91%, respectively, compared to the control.

## 3. Discussion

### 3.1. Effect of Applying 6-BA on Grain Yield and Filling Process

Among the various field management practices, the application of exogenous hormones is an excellent approach to improving crop production. In previous studies, exogenous 6-BA could promote endosperm cell proliferation and accelerate storage material accumulation by altering endogenous hormones levels in the grains, thereby increasing grain weight and yield in maize under waterlogging stress [46] and in wheat under heat stress [47]. This study showed that applying 6-BA had a positive effect on maize yield under dense planting conditions by improving grain filling characteristics, enhancing the activities of critical enzymes for starch synthesis, and altering endogenous hormones levels in the grains. Among the yield components, 6-BA application significantly improved 1000-grain weight over the control under dense planting conditions, but showed no significant effect on ear number or grains per ear in either variety. These results suggested 6-BA application mainly increased grain weight to improve maize yield under dense planting conditions.

Generally, the grain filling process is controlled by filling rate and duration, or a combination of the two [48]. In the current study, both the maximum and average grain filling rates were lower in IG compared to SG, but active grain filling duration was not significantly different between them. These results suggested that IG had a poorer filling process, mainly due to a slower filling rate. Therefore, grain weight was significantly lower in IG compared to SG, in agreement with previous results [15,16]. In wheat, applying 6-BA accelerated the grain filling rate and prolonged the active filling duration, thereby improving grain weight under waterlogging and shading stress [49]. Gao et al. showed that applying 6-BA improved maize grain weight mainly by increasing the rates of endosperm cell proliferation and grain filling [41]. Our results showed that the application of 6-BA increased the maximum and average grain filling rates in IG and SG of both varieties without noticeably affecting the active filling duration. This showed that the increase in grain weight after 6-BA application under dense planting conditions was mainly due to a higher filling rate. Notably, the increase in grain weight was greater in IG than in SG, suggesting that IG was more sensitive to exogenous 6-BA. Wei et al. also reported that IG in maize showed greater sensitivity to density and nitrogen application rate than SG [50]. Meanwhile, in the presence of inappropriate cultivation practices or environmental stress, the development of IG is more likely to be inhibited than that of SG, even to the point of abortion [51,52]. All these results suggested that IG may be more sensitive to environmental variables than SG in maize, and this phenomenon is also observed in wheat [53] and rice [54]. Therefore, using appropriate agronomic practices to promote IG filling will be key to further improving grain yield.

### 3.2. Effect of Applying 6-BA on Grain Starch Accumulation 

Since starch represents about 70% of maize grain dry weight, starch synthesis and accumulation are primarily responsible for grain filling. Maize starch is composed of amylose and amylopectin, whose ratio and concentration in the grains determine starch quality [55]. Our results showed that IG contained significantly lower contents of amylose, amylopectin, and total starch than SG, resulting in grain weight differences between the two. 6-BA is considered to be one of the most potent plant hormones in terms of increasing biomass and starch accumulation [56]. In wheat grains, 6-BA application promotes starch granule production and growth, while stimulating starch accumulation [49]. Similar to grain weight in this study, applying 6-BA increased amylose, amylopectin, and total starch contents in maize grains compared to the control, with greater increases in IG than SG. 

Several enzymes are responsible for catalyzing starch synthesis. SuSy is primarily responsible for mobilizing sucrose to starch, while AGPase is an essential enzyme that limits the rate of starch formation [57]. GBSS is mainly involved in amylose synthesis, whereas SSS and SBE are mainly required for amylopectin synthesis [58]. Increasing these enzyme activities during maize grain development could accelerate starch accumulation and thus increase grain weight [23,59]. In this study, SuSy, AGPase, GBSS, SSS, and SBE activities all varied along an unimodal curve, in agreement with our previous research [60]. We also found a significant positive relationship between the activities of all the above enzymes and the contents of amylose, amylopectin, and total starch (Table 3). Meanwhile, all of the above enzyme activities were reduced in IG compared to SG, consistent with our previous proteomic studies showing that several starch synthesis-related proteins were underexpressed in IG compared to SG [61]. Therefore, the lower starch content and poorer grain filling in IG should be strongly associated with lower activity of crucial enzymes for starch synthesis. Further research could use molecular biology to regulate these enzymes for grain filling. Previous research has shown that 6-AB application can affect starch accumulation by regulating enzyme activities in crop grains. In rice grains, applying 6-BA enhanced SuSy, AGPase, and SSS activities, thereby reducing the negative effect of excess nitrogen on starch accumulation [62]. Luo et al. showed that the application of 6-BA preserved starch and sucrose levels in lotus seeds by controlling relevant synthetic or degrading enzymes [63]. Our results showed that applying 6-BA enhanced SuSy, AGPase, GBSS, SSS, and SBE activities in maize grains under high planting density. Correspondingly, the amylose, amylopectin, and total starch contents increased. Our research also discovered that these key enzyme activities were more responsive to exogenous 6-BA in IG than in SG, which helps to explain why starch content and grain weight increased more in IG than in SG following 6-BA application. Previous studies have reported that the application of exogenous hormones can increase the activities of enzymes associated with starch synthesis by upregulating the expression of related genes in rice [64] or proteins in potato [65]. In this study, the application of 6-BA may have increased the expression of genes or proteins related to SuSy, AGPase, SSS, GBSS, and SBE, thereby enhancing the activities of these enzymes and promoting starch accumulation. However, further investigations are needed to explore the potential regulatory mechanisms of these enzymes associated with starch synthesis in maize grains positioned at different ear locations after 6-BA application under high-density conditions. In conclusion, applying 6-BA could accelerate starch accumulation by enhancing the activities of relevant enzymes under dense planting conditions, which would be beneficial for improving grain filling and increasing grain weight.

### 3.3. Effect of Applying 6-BA on Grain Endogenous Hormones 

As key regulators of cell proliferation, differentiation, and substance accumulation during crop grain development, plant endogenous hormones play an important role in determining grain sink capacity and strength [33,38,46,66]. Our results showed a general unimodal curve for IAA, ZR, and ABA between 10 and 50 DAP, peaking at 20 DAP, while GA_3_ displayed a decreasing trend in the grains. Meanwhile, ZR, IAA, and ABA levels were significantly positively correlated with maximum and mean grain filling rates, but the GA_3_ level was significantly negatively correlated (Table 4), in agreement with previous studies [67]. We also noticed that the levels of ZR, IAA, and ABA were much lower in IG than in SG. ZR and IAA have been suggested to be essential in regulating early grain development and their higher levels may enhance sink capacity and strength by stimulating endosperm cell proliferation and growth [68,69]. ABA is strongly correlated with grain development, and its accumulation in grains may accelerate the grain filling rate by stimulating storage material revitalization [70]. Consequently, lower ZR, IAA, and ABA levels in IG may contribute to smaller sink capacity and weaker strength, ultimately causing lower grain filling rate and weight. In contrast to IAA, ZR, and ABA, the GA_3_ level of IG was significantly higher than that of SG, in line with prior research [49,67]. However, this may be detrimental to starch synthesis and accumulation in IG, as high levels of GA_3_ promote starch catabolism by increasing the activity of a-amylase and other hydrolases [71,72]. Taken together, our findings appear to further confirm that endogenous hormone dysregulation is one of the major contributors to the less favorable filling process in maize IG. 

Previous work has suggested that applying 6-BA can mitigate the disruption of endogenous hormones levels in grains by abiotic stresses, thereby improving grain filling and weight [46,47]. More importantly, 6-BA application regulates the overall balance of endogenous hormones rather than just the level of one hormone in grains [41,46]. Our research showed that applying 6-BA did not change the overall trend of ZR, IAA, ABA, and GA_3_, but increased IAA, ZR, and ABA levels and decreased the GA_3_ level. This would accelerate the grain filling rate by improving grain sink capacity and strength, thereby promoting storage accumulation and ultimately increasing grain weight [40,46,47]. A previous study found that endogenous hormones levels in maize IG were more sensitive to changes in density and nitrogen application rate than in SG [50]. The same strong response of endogenous hormones in IG compared to SG after 6-BA application was observed in our study, which may also be an important factor for a better effect of 6-BA application in improving the filling process of IG. Clearly, applying 6-BA can also improve the grain filling process by altering endogenous hormones levels. Many studies have shown that exogenous hormones can alter endogenous hormone levels by modulating a variety of related synthetic and metabolic genes and enzymes [73]. Panda et al. showed that exogenous 6-BA regulated cytokinin levels by modulating cytokinin oxidase activity and the expression of cell cycle regulators and cytokinin signaling components in rice grains [74]. In this study, the application of 6-BA may have regulated the genes and enzymes that control endogenous hormone biosynthesis, metabolism, and signalling, resulting in changes in endogenous hormone levels in maize grains. However, further studies are needed on the detailed regulatory mechanisms of interactions between exogenous 6-BA and dynamic variations of endogenous hormones in maize grains placed at different ear positions under high-density conditions.

## 4. Materials and Methods

### 4.1. Test Site and Conditions

**Figure 5:**
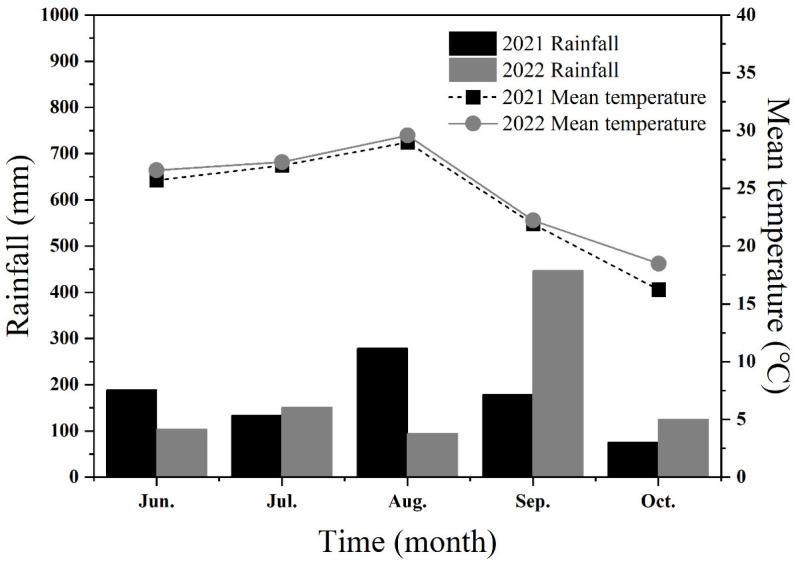
The average temperature and rainfall information during the maize growing season in 2021 and 2022.

The field test was conducted at the farm of Shandong Agricultural University, China (36°10′ N, 117°04′ E) during 2021 and 2022. Information on average temperature and rainfall recorded during the maize growing season for both years is shown in Figure 5. The test area is a typical brown loam type with 11.41 g kg^−1^ organic matter, 58.92 mg kg^−1^ available nitrogen, 42.83 mg kg^−1^ available phosphate, and 76.85 mg kg^−1^ exchangeable potassium in the top 20 cm of the soil before the experiment. These indicators were determined according to the soil agrochemical analysis protocol of Bao [75]: organic matter by potassium dichromate capacity method, available nitrogen by alkaline solution diffusion method, available phosphate by sodium bicarbonate extraction colorimetry method, and exchangeable potassium by ammonium acetate flame photometry method.

### 4.2. Experimental Design and Sampling

Denghai 605 (DH605, FAO 100) and Zhengdan 958 (ZD958, FAO 100) were used as test materials. Both varieties have a growing period of approximately 102 days and are widely grown in China. The recommended local planting density for both varieties is 75,000 plants ha^−1^. Seed of both varieties was supplied by China National Seeds Group Co., Ltd., Beijing, China. In both years, a high density of 90,000 plants ha^−1^ was sown on 14 June. At the flowering stage, 6-BA was applied uniformly to the surface of the maize leaves with a sprayer, and the control group was sprayed with water. 6-BA was purchased from Beijing Solarbio Science & Technology Co., Ltd., Beijing, China. 6-BA and water were sprayed on three consecutive days from 16:00 to 18:00. Based on previous studies [46], 6-BA was sprayed at a dose of 100 mg L^−1^ and a rate of 150 ± 5 mL per plant. All solutions were finalized with 0.5% (*v*/*v*) of Tween-20 as a surfactant. Three replicates of each treatment were employed in a fully randomized design. Each plot measured 12 m by 3 m and had 5 rows, and the spaces between the rows were 60 cm. Each plot received 280 kg ha^−1^ N (urea), 100 kg ha^−1^ P_2_O_5_ (calcium superphosphate), and 200 kg ha^−1^ K_2_O (potassium sulfate) fertilizer. All of the phosphorus, potassium, and half of the nitrogen fertilizer were applied before sowing, and the additional half of the nitrogen fertilizer was applied at the jointing stage.

At least 100 healthy and uniformly growing plants per plot were marked at the tasseling stage, and artificial pollination was carried out to ensure consistency of pollination. For each treatment, other management practices such as irrigation and treatment of weeds, diseases, and pests were adequately controlled. In each plot, ears from five marked plants were collected at 10-day intervals between 10 and 50 days after pollination (DAP). Each ear was then divided equally into upper, middle, and lower halves, and the upper and middle grains were selected as IG and SG, respectively. Some 50% of the grains were immediately frozen in liquid nitrogen and then stored at −80 °C to assay starch synthesis critical enzyme activity and endogenous hormones levels, while the remaining grains were baked at 105 °C for 30 min and then dried at 80 °C to a constant weight to assess grain filling characteristics and starch content.

### 4.3. Test Items and Methods

#### 4.3.1. Grain Filling Process

Some 100 grains of IG and SG were collected to determine grain dry weight. The grain filling process was fitted by logistic equations according to Yin et al. [76]:

y = A/(1 + Be − Ct)(1)

The filling parameters were then evaluated using the following equations:

W_max_ = A/2 (2)

G_max_ = (C × W_max_) × [1 − (W_max_/A)] (3)

G_ave_ = (95% of A − 5% of A)/(t_2_ − t_1_) (4)

P = 6/C(5)

In the equation, y stands for grain weight, t for days after pollination, A for final grain weight, and B and C for coefficients derived by regression. t_1_ and t_2_ refer to the days when 5% and 95% of A are reached, respectively. W_max_ is grain weight of reaching maximum grain filling rate; G_max_ is maximum grain filling rate; G_ave_ is average grain filling rate; and P is active grain filling duration.

#### 4.3.2. Starch Content and Starch Synthesis Critical Enzyme Activity

The amylose and amylopectin contents were quantified using the “double-wave-length” approach recommended by Zhu et al. [77]. The primary and special wavelengths employed for the determination of amylase and amylopectin content were 556 and 737 nm, and 620 and 479 nm, respectively. Total starch content was calculated by combining amylose and amylopectin content. SuSy, AGPase, GBSS, SSS, and SBE activities were quantified using the appropriate test kit supplied by Beijing Solarbio Science & Technology Co., Ltd., Beijing, China. Three biological replicates were performed for each enzyme activity assay.

#### 4.3.3. Endogenous Hormone Levels

ZR, IAA, ABA, and GA_3_ were extracted with 80% (*v*/*v*) methanol and then analyzed by high-performance liquid chromatography (LC-10 AD, Shimadzu, Japan), as described by Sun et al. [78]. Standards for each hormone were purchased from Beijing Solarbio Science & Technology Co., Ltd., Beijing, China. A calibration curve was created for each hormone using standards with concentrations between 0 and 0.2 mg mL^−1^. Three biological replicates were performed for each endogenous-hormone-level assay.

#### 4.3.4. Yield and Yield Components

Yield (moisture content was 14%), ear number, grains per ear, and 1000-grain weight were all calculated after harvesting 30 ears from the central region of each plot when they had reached physiological maturity.

### 4.4. Statistical Analysis

Statistical analyses were performed using SPSS version 17.0 (SPSS Inc., Chicago, IL, USA). Data from each sampling date were analyzed separately. Data were first checked for normality (Kolmogorov–Smirnov test) and homogeneity of variance (Bartlett-Box test). The data had a normal distribution and homogeneous variance. Significant differences between different treatments were assessed using Duncan’s test (*p* = 0.05). Pearson correlation analyses were also applied. Figures in the article were plotted using Sigma Plot version 12.0 (SYSTAT Inc., San Jose, CA, USA).

## 5. Conclusions

Lower activities of critical enzymes for starch synthesis and imbalanced endogenous hormone levels may be important reasons for the poorer filling process in IG, resulting in significantly lower starch content and grain weight than in SG. An improvement in grain filling and an increase in final grain weight and yield of maize under crowded conditions was achieved as a result of applying 6-BA, which improved filling characteristics, accelerated starch accumulation by enhancing the activities of critical enzymes for starch synthesis, and altered endogenous hormone levels in the grains. In addition, IG showed a greater response to exogenous 6-BA than SG, indicating that using agronomic practices to promote IG filling is an important approach to further improve maize yield under dense planting conditions. However, the potential regulatory mechanisms of exogenous 6-BA on the activities of enzymes associated with starch synthesis and the levels of endogenous hormones in maize grains at different ear positions were not investigated in depth in this study. In the future, traditional and novel molecular biology techniques can be applied to further investigate these issues. Taken together, our research is important in guiding the use of exogenous hormones to improve maize yield under high-density conditions.

## Figures and Tables

**Table 1:** Effects of applying 6-BA on maize yield and yield components in 2021 and 2022.

Year	Variety	Treatments	Ear Number (Ears hm^−2^)	Grains per Ear	1000-Grain Weight (g)	Yield (kg hm^−2^)
2021	DH605	CK	84,147.36 ± 214 a	434.41 ± 7.63 a	352.73 ± 3.14 c	12,893.81 ± 439 b
		6-BA	84,138.38 ± 304 a	435.49 ± 3.46 a	380.75 ±4.53 a	13,951.43 ± 130 a
	ZD958	CK	84,143.11 ± 488 a	447.59 ± 6.67 a	346.70 ± 3.67 c	13,003.87 ± 237 b
		6-BA	84,558.38 ± 225 a	443.80 ± 16.79 a	370.09 ± 5.11 b	13,884.28 ± 454 a
2022	DH605	CK	83,893.33 ± 101 a	451.72 ± 17.99 a	357.26 ± 3.03 c	13,538.95 ± 563 bc
		6-BA	83,903.47 ± 131 a	453.04 ± 3.85 a	384.75 ± 5.73 a	14,625.20 ± 149 a
	ZD958	CK	84,473.11 ± 581 a	448.98 ± 6.64 a	347.70 ± 5.73 d	13,185.40 ± 268 c
		6-BA	84,948.38 ± 221 a	451.98 ± 3.51 a	366.09 ±4.84 b	14,055.86 ± 489 ab

CK and 6-BA correspond to maize plants treated with water and 6-BA, respectively. Data are shown as mean ± S.D. (*n* = 3). Values in the same column with different small letters are significantly different within the year according to Duncan’s test (*p* = 0.05).

**Table 2:** Effects of applying 6-BA on grain filling characteristics (2022).

Variety	Treatments	W_max_ (g 100 Grains^−1^)	G_max_ (g 100 Grains^−1^ d^−1^)	G_ave_ (g 100 Grains^−1^ d^−1^)	P (d)
DH605	ICK	10.89 ± 0.41 d	0.73 ± 0.06 d	0.44 ± 0.02 d	44.60 ± 2.57 a
	I6-BA	12.70 ± 0.40 c	0.87 ± 0.04 c	0.52 ± 0.03 c	44.02 ± 4.50 a
	SCK	14.51 ± 0.90 b	1.01 ± 0.03 b	0.60 ± 0.03 b	43.23 ± 6.00 a
	S-6BA	16.57 ± 0.55 a	1.13 ± 0.07 a	0.68 ± 0.02 a	43.87 ± 8.20 a
ZD958	ICK	11.59 ± 0.26 d	0.82 ± 0.04 d	0.49 ± 0.03 d	42.62 ± 4.21 a
	I6-BA	12.54 ± 0.35 c	0.94 ± 0.03 c	0.56 ± 0.01 c	40.24 ± 6.82 a
	SCK	14.36 ± 0.30 b	1.04 ± 0.05 b	0.62 ± 0.03 b	41.40 ± 5.05 a
	S6-BA	15.29 ± 0.21 a	1.14 ± 0.02 a	0.68 ± 0.01 a	40.32 ± 6.47 a

ICK and I6-BA correspond to inferior grains treated with water and 6-BA, respectively. SCK and S6-BA correspond to superior grains treated with water and 6-BA, respectively. Data are shown as mean ± S.D. (*n* = 3). Values in the same column with different small letters are significantly different within the same variety according to Duncan’s test (*p* = 0.05). W_max_, grain weight of reaching maximum grain filling rate; G_max_, maximum grain filling rate; G_ave_, average grain filling rate; P, active grain filling duration.

**Table 3:** Correlation analysis between starch content and starch synthesis critical enzyme activity.

	SuSy	AGPase	GBSS	SSS	SBE
Amylose	0.931 **	0.751 *	0.899 **	0.810 **	0.793 *
Amylopectin	0.954 **	0.819 **	0.835 **	0.928 **	0.961 **
Total starch	0.951 **	0.804 **	0.885 **	0.931 **	0.965 **

SuSy, sucrose synthase; AGPase, ADP-glucose pyrophosphorylase; GBSS, granule-bound starch synthase; SSS, soluble starch synthase; SBE, starch branching enzyme. Significant differences are shown by * and ** at the 0.05 and 0.01 probability levels (*n* = 8), respectively.

**Table 4:** Correlation analysis between endogenous hormones levels and grain filling parameters.

	W_max_	G_max_	G_ave_	P
ZR	0.817 *	0.860 **	0.864 **	0.422
IAA	0.874 **	0.963 **	0.962 **	0.608
ABA	0.855 **	0.946 **	0.944 **	0.623
GA_3_	−0.832 *	−0.884 **	−0.886 **	−0.464

ZR, zeatin riboside; IAA, indole-3-acetic acid; ABA, abscisic acid; GA_3_, gibberellin; W_max_, grain weight of reaching maximum grain filling rate; G_max_, maximum grain filling rate; G_ave_, average grain filling rate; P, active grain filling duration. Significant differences are shown by * and ** at the 0.05 and 0.01 probability levels (*n* = 8), respectively.

## Data Availability

The corresponding author can provide the data backing up these conclusions upon reasonable request.

## References

[1] Ten Berge H.F.M., Hijbeek R., Van Loon M.P., Van Loon J., Rurinda K., Tesfaye S., Zingore P., Craufurd J., Van Heerwaarden F., Brentrup J.J. (2019). Maize crop nutrient input requirements for food security in sub-Saharan Africa. Glob. Food Secur..

[2] Erenstein O., Jaleta M., Sonder K., Mottaleb K., Prasanna B.M. (2022). Global maize production, consumption and trade: Trends and R&D implications. Food Secur..

[3] Zhou B., Yue Y., Sun X., Ding Z., Ma W., Zhao M. (2017). Maize kernel weight responses to sowing date-associated variation in weather conditions. Crop J..

[4] Li Q., Du L., Feng D., Ren Y., Li Z., Kong F., Yuan J. (2020). Grain-filling characteristics and yield differences of maize cultivars with contrasting nitrogen efficiencies. Crop J..

[5] Shi D., Li Y., Zhang J., Liu P., Zhao B., Dong S. (2016). Increased plant density and reduced N rate lead to more grain yield and higher resource utilization in summer maize. J. Integr. Agric..

[6] Testa G., Reyneri A., Blandino M. (2016). Maize grain yield enhancement through high plant density cultivation with different inter-row and intra-row spacings. Eur. J. Agron..

[7] Zhang G., Shen D., Xie R., Ming B., Hou P., Xue J., Li R., Chen J., Wang K., Li S. (2020). Optimizing planting density to improve nitrogen use of super high yield maize. Agron. J..

[8] Li J., Xie R.Z., Wang K.R., Ming B., Guo Y.Q., Zhang G.Q., Li S.K. (2015). Variations in maize dry matter, harvest index, and grain yield with plant density. Agron. J..

[9] Assefa Y., Vara Prasad P.V., Carter P., Hinds M., Bhalla G., Schon R., Jeschke M., Paszkiewicz S., Ciampitti I.A. (2016). Yield responses to planting density for US modern corn hybrids: A synthesis-analysis. Crop Sci..

[10] Jia Q., Sun L., Mou H., Ali S., Liu D., Zhang Y., Zhang P., Ren X., Jia Z. (2018). Effects of planting patterns and sowing densities on grain–filling, radiation use efficiency and yield of maize (*Zea mays* L.) in semi-arid regions. Agric. Water Manag..

[11] Yan S., Wu Y., Fan J., Zhang F., Qiang S., Zheng J., Xiang Y., Guo J., Zou H. (2019). Effects of water and fertilizer management on grain filling characteristics, grain weight and productivity of drip-fertigated winter wheat. Agric. Water Manag..

[12] Ordóñez R.A., Savin R., Cossani C.M., Slafer G.A. (2018). Maize grain weight sensitivity to source-sink manipulations under a wide range of field conditions. Crop Sci..

[13] Liu K., Harrison M.T., Yan H., Liu D.L., Meinke H., Hoogenboom G., Wang B., Peng B., Guan K., Jaegermeyr J. (2023). Silver lining to a climate crisis in multiple prospects for alleviating crop waterlogging under future climates. Nat. Commun..

[14] Zhao F., Jing L., Wang D., Bao F., Lu W., Wang G. (2018). Grain and starch granule morphology in superior and inferior kernels of maize in response to nitrogen. Sci. Rep..

[15] Liu D.Y., Zhang W., Liu Y.M., Chen X.P., Zou C.Q. (2020). Soil application of zinc fertilizer increases maize yield by enhancing the kernel number and kernel weight of inferior grains. Front. Plant Sci..

[16] Zhai L., Wang Z., Song S., Zhang L., Zhang Z., Jia X. (2021). Tillage practices affects the grain filling of inferior kernel of summer maize by regulating soil water content and photosynthetic capacity. Agric. Water Manag..

[17] Zhu D., Fang C., Qian Z., Guo B., Huo Z. (2021). Differences in starch structure, physicochemical properties and texture characteristics in superior and inferior grains of rice varieties with different amylose contents. Food Hydrocolloid..

[18] Xu H., Cai T., Wang Z., He M. (2015). Physiological basis for the differences of productive capacity among tillers in winter wheat. J. Integr. Agric..

[19] Peng T., Sun H., Qiao M., Zhao Y., Du Y., Zhang J., Li J., Tang G., Zhao Q. (2014). Differentially expressed microRNA cohorts in seed development may contribute to poor grain filling of inferior spikelets in rice. BMC Plant Biol..

[20] Bonelli L.E., Monzon J.P., Cerrudo A., Rizzalli R.H., Andrade F.H. (2016). Maize grain yield components and source-sink relationship as affected by the delay in sowing date. Field Crops Res..

[21] Yang H., Gu X., Ding M., Lu W., Lu D. (2018). Heat stress during grain filling affects activities of enzymes involved in grain protein and starch synthesis in waxy maize. Sci. Rep..

[22] Huang L.C., Tan H.Y., Zhang C.Q., Li Q.F., Liu Q.Q. (2021). Starch biosynthesis in cereal endosperms: An updated review over the last decade. Plant Commun..

[23] Liu X.M., Gu W.R., Li C.F., Jing L.I., Wei S. (2021). Effects of nitrogen fertilizer and chemical regulation on spring maize lodging characteristics, grain filling and yield formation under high planting density in Heilongjiang Province, China. J. Integr. Agric..

[24] Yuan C., Wang S., Lu D. (2022). Fertilization time of slow-release fertilizer affects the physicochemical properties of starch from spring-sown waxy maize. J. Sci. Food Agric..

[25] Ahmad I., Kamran M., Meng X., Ali S., Bilegjargal B., Cai T., Liu T., Han Q. (2019). Effects of plant growth regulators on seed filling, endogenous hormone contents and maize production in semiarid regions. J. Plant Growth Regul..

[26] Liu Y., Gu D., Wu W., Wen X., Liao Y. (2013). The relationship between polyamines and hormones in the regulation of wheat grain filling. PLoS ONE.

[27] Xu Y., Li K., Zhu K., Tian Y., Yu Q., Zhang W., Wang Z. (2020). Effect of exogenous plant hormones on agronomic and physiological performance of a leaf early-senescent rice mutant osled. Plant Growth Regul..

[28] Yang J., Zhang J., Huang Z., Wang Z., Zhu Q., Liu L. (2002). Correlation of cytokinin levels in the endosperms and roots with cell number and cell division activity during endosperm development in rice. Ann. Bot..

[29] Lur H.S., Setter T.L. (1993). Role of auxin in maize endosperm development (timing of nuclear DNA endoreduplication, zein expression, and cytokinin). Plant Physiol..

[30] Shi C.L., Dong N.Q., Guo T., Ye W.W., Shan J.X., Lin H.X. (2020). A quantitative trait locus *GW6* controls rice grain size and yield through the gibberellin pathway. Plant J..

[31] Seiler C., Harshavardhan V.T., Rajesh K., Reddy P.S., Strickert M., Rolletschek H., Scholz U., Wobus U., Sreenivasulu N. (2011). ABA biosynthesis and degradation contributing to ABA homeostasis during barley seed development under control and terminal drought-stress conditions. J. Exp. Bot..

[32] Qin S., Zhang Z., Ning T., Ren S., Su L., Li Z. (2013). Abscisic acid and aldehyde oxidase activity in maize ear leaf and grain relative to post-flowering photosynthetic capacity and grain-filling rate under different water/nitrogen treatments. Plant Physiol. Biochem..

[33] Liu Y., Sui Y., Gu D., Wen X., Chen Y., Li C., Liao Y. (2013). Effects of conservation tillage on grain filling and hormonal changes in wheat under simulated rainfall conditions. Field Crops Res..

[34] Jing F.U., Xu Y.J., Chen L., Yuan L.M., Wang Z.Q., Yang J.C. (2013). Changes in enzyme activities involved in starch synthesis and hormone concentrations in superior and inferior spikelets and their association with grain filling of super rice. Rice Sci..

[35] Lv X., Han J., Liao Y., Liu Y. (2017). Effect of phosphorus and potassium foliage application post-anthesis on grain filling and hormonal changes of wheat. Field Crops Res..

[36] Cai T., Xu H., Peng D., Yin Y., Yang W., Ni Y., Chen X., Xu C., Yang D., Cui Z. (2014). Exogenous hormonal application improves grain yield of wheat by optimizing tiller productivity. Field Crops Res..

[37] Wang L., Yan Y., Lu W., Lu D. (2020). Application of exogenous phytohormones at silking stage improve grain quality under post-silking drought stress in waxy maize. Plants.

[38] Li G., Liang Z., Li Y., Liao Y., Liu Y. (2020). Exogenous spermidine regulates starch synthesis and the antioxidant system to promote wheat grain filling under drought stress. ACTA Physiol. Plant..

[39] Zhang H., Horgan K.J., Reynolds P.H.S., Jameson P.E. (2010). 6-Benzyladenine metabolism during reinvigoration of mature *Pinus radiata* buds in vitro. Tree Physiol..

[40] Ren B., Zhu Y., Zhang J., Dong S., Liu P., Zhao B. (2016). Effects of spraying exogenous hormone 6-benzyladenine (6-BA) after waterlogging on grain yield and growth of summer maize. Field Crops Res..

[41] Gao Z., Liang X.G., Zhang L., Lin S., Zhao X., Zhou L.L., Shen S., Zhou S.L. (2017). Spraying exogenous 6-benzyladenine and brassinolide at tasseling increases maize yield by enhancing source and sink capacity. Field Crops Res..

[42] Ghorbani Javid M., Sorooshzadeh A., Modarres Sanavy S.A.M., Allahdadi I., Moradi F. (2011). Effects of the exogenous application of auxin and cytokinin on carbohydrate accumulation in grains of rice under salt stress. Plant Growth Regul..

[43] Akter N., Rafiqul Islam M., Abdul Karim M., Hossain T. (2014). Alleviation of drought stress in maize by exogenous application of gibberellic acid and cytokinin. J. Crop Sci. Biotechnol..

[44] Hu J., Ren B., Dong S., Liu P., Zhao B., Zhang J. (2020). Comparative proteomic analysis reveals that exogenous 6-benzyladenine (6-BA) improves the defense system activity of waterlogged summer maize. BMC Plant Biol..

[45] Wang S., Yang S., He D., Yi Y., Fu Y., Yin D., Zhao H., Xiao C. (2022). Exogenous 6-benzyladenine treatment alleviates cold stress in early *japonica* rice at booting in Northeast China. Agron. J..

[46] Ren B., Hu J., Zhang J., Dong S., Liu P., Zhao B. (2019). Spraying exogenous synthetic cytokinin 6-benzyladenine following the waterlogging improves grain growth of waterlogged maize in the field. J. Agron. Crop Sci..

[47] Yang D., Li Y., Shi Y., Cui Z., Luo Y., Zheng M., Chen J., Li Y., Yin Y., Wang Z. (2016). Exogenous cytokinins increase grain yield of winter wheat cultivars by improving stay-green characteristics under heat stress. PLoS ONE.

[48] Hammad H.M., Abbas F., Ahmad A., Bakhat H.F., Farhad W., Wilkerson C.J., Fahad S., Hoogenboom G. (2020). Predicting kernel growth of maize under controlled water and nitrogen applications. Int. J. Plant Prod..

[49] Zhang W., Wang B., Zhang A., Zhou Q., Li Y., Li L., Ma S., Fan Y., Huang Z. (2022). Exogenous 6-benzylaminopurine enhances waterlogging and shading tolerance after anthesis by improving grain starch accumulation and grain filling. Front. Plant Sci..

[50] Wei S., Wang X., Li G., Qin Y., Jiang D., Dong S. (2019). Plant density and nitrogen supply affect the grain-filling parameters of maize kernels located in different ear positions. Front. Plant Sci..

[51] Shen S., Liang X.G., Zhang L., Zhao X., Liu Y.P., Lin S., Gao Z., Wang P., Wang Z.M., Zhou S.L. (2020). Intervening in sibling competition for assimilates by controlled pollination prevents seed abortion under postpollination drought in maize. Plant Cell Environ..

[52] Shen S., Zhang L., Liang X.G., Zhao X., Lin S., Qu L.H., Liu Y.P., Gao Z., Ruan Y.L., Zhou S.L. (2018). Delayed pollination and low availability of assimilates are major factors causing maize kernel abortion. J. Exp. Bot..

[53] Liang W., Zhang Z., Wen X., Liao Y., Liu Y. (2017). Effect of non-structural carbohydrate accumulation in the stem pre-anthesis on grain filling of wheat inferior grain. Field Crops Res..

[54] Zhao C., Liu G., Chen Y., Jiang Y., Shi Y., Zhao L., Liao P., Wang W., Xu K., Dai Q. (2022). Excessive nitrogen application leads to lower rice yield and grain quality by inhibiting the grain filling of inferior grains. Agriculture.

[55] Chung K.H., Othman Z., Lee J.S. (2015). Gamma irradiation of corn starches with different amylose-to-amylopectin ratio. J. Food Sci. Technol..

[56] Liu Y., Chen X., Wang X., Fang Y., Zhang Y., Huang M., Zhao H. (2019). The influence of different plant hormones on biomass and starch accumulation of duckweed: A renewable feedstock for bioethanol production. Renew. Energy.

[57] Hannah L.C., James M. (2008). The complexities of starch biosynthesis in cereal endosperms. Curr. Opin. Biotechnol..

[58] James M.G., Denyer K., Myers A.M. (2003). Starch synthesis in the cereal endosperm. Curr. Opin. Plant Biol..

[59] Nelson O., Pan D. (1995). Starch synthesis in maize endosperms. Annu. Rev. Plant Biol..

[60] Yu T., Li G., Dong S., Liu P., Zhang J., Zhao B. (2016). Proteomic analysis of maize grain development using iTRAQ reveals temporal programs of diverse metabolic processes. BMC Plant Boil..

[61] Yu T., Li G., Liu P., Dong S., Zhang J., Zhao B. (2017). Proteomics analysis of maize (*Zea mays* L.) grain based on iTRAQ reveals molecular mechanisms of poor grain filling in inferior grains. Plant Physiol. Biochem..

[62] Chen Y., Teng Z., Yuan Y., Yi Z., Zheng Q., Yu H., Lv J., Wang Y., Duan M., Zhang J. (2022). Excessive nitrogen in field-grown rice suppresses grain filling of inferior spikelets by reducing the accumulation of cytokinin and auxin. Field Crops Res..

[63] Luo S., Hu H., Zhang L., Zhou H., Li P. (2017). Sugars in postharvest lotus seeds were modified by 6-benzylaminopurine treatment through altering related enzymes involved in starch-sucrose metabolism. Sci. Hortic..

[64] Zhu G., Ye N., Yang J., Peng X., Zhang J. (2011). Regulation of expression of starch synthesis genes by ethylene and ABA in relation to the development of rice inferior and superior spikelets. J. Exp. Bot..

[65] Cheng L., Wang D., Wang Y., Xue H., Zhang F. (2020). An integrative overview of physiological and proteomic changes of cytokinin-induced potato (*Solanum tuberosum* L.) tuber development in vitro. Physiol. Plantarum..

[66] Gao J., Shi J., Dong S., Liu P., Zhao B., Zhang J. (2018). Grain development and endogenous hormones in summer maize (*Zea mays* L.) submitted to different light conditions. Int. J. Biometeorol..

[67] Xu Y., Gu D., Zhang B., Zhang H., Wang Z., Yang J. (2013). Hormone contents in kernels at different positions on an ear and their relationship with endosperm development and kernel filling in maize. Acta Agron. Sin..

[68] Yang J., Zhang J., Wang Z., Zhu Q., Liu L. (2002). Abscisic acid and cytokinins in the root exudates and leaves and their relationship to senescence and remobilization of carbon reserves in rice subjected to water stress during grain filling. Planta.

[69] Xu G., Zhang J., Lam H.M., Wang Z., Yang J. (2007). Hormonal changes are related to the poor grain filling in the inferior spikelets of rice cultivated under non-flooded and mulched condition. Field Crops Res..

[70] Seo M., Koshiba T. (2002). Complex regulation of ABA biosynthesis in plants. Trends Plant Sci..

[71] Pharis R.P., King R.W. (1985). Gibberellins and reproductive development in seed plants. Annu. Rev. Plant Physiol..

[72] Liu Y., Han J., Liu D., Gu D., Wang Y., Liao Y., Wen X. (2015). Effect of plastic film mulching on the grain filling and hormonal changes of maize under different irrigation conditions. PLoS ONE.

[73] Kosakivska I.V., Vedenicheva N.P., Babenko L.M., Voytenko L.V., Romanenko K.O., Vasyuk V.A. (2022). Exogenous phytohormones in the regulation of growth and development of cereals under abiotic stresses. Mol. Biol. Rep..

[74] Panda B.B., Sekhar S., Dash S.K., Behera L., Shaw B.P. (2018). Biochemical and molecular characterisation of exogenous cytokinin application on grain filling in rice. BMC Plant Boil..

[75] Bao S.D. (2000). Soil Agro-Chemistrical Analysis.

[76] Yin X., Goudriaan J.A.N., Lantinga E.A., Vos J.A.N., Spiertz H.J. (2003). A flexible sigmoid function of determinate growth. Ann. Bot..

[77] Zhu T., Jackson D.S., Wehling R.L., Geera B. (2008). Comparison of amylose determination methods and the development of a dual wavelength iodine binding technique. Cereal Chem..

[78] Sun J., Wang H., Ren H., Zhao B., Zhang J., Ren B., Liu P. (2023). Maize (*Zea mays* L.) responses to heat stress: Mechanisms that disrupt the development and hormone balance of tassels and pollen. J. Agron. Crop Sci..

